# An implementation science study of a campus-based drug checking service at a Canadian university

**DOI:** 10.1371/journal.pone.0332899

**Published:** 2025-09-18

**Authors:** Lauren Airth, Bruce Wallace, Mary Clare Kennedy, Lucas Standing, Antoine Marcheterre, Lianping Ti, Jennifer Matthews, Melissa Feddersen, Nelly D. Oelke

**Affiliations:** 1 School of Nursing, University of British Columbia, Kelowna, British Columbia, Canada; 2 Hein lab, University of British Columbia, Vancouver, British Columbia, Canada; 3 British Columbia Centre on Substance Use, Vancouver, British Columbia, Canada; 4 Canadian Institute for Substance Use Research, University of Victoria, Victoria, British Columbia, Canada; 5 School of Social Work, University of Victoria, Victoria, British Columbia, Canada; 6 School of Social Work, University of British Columbia, Kelowna, British Columbia, Canada; 7 Interior Health, Kamloops, British Columbia, Canada; 8 Department of Medicine, University of British Columbia, Vancouver, British Columbia, Canada; 9 Campus Wellness and Education, University of British Columbia, Kelowna, British Columbia, Canada; 10 Rural Coordination Centre of British Columbia, Vancouver, British Columbia, Canada; University of Science and Technology of Fujairah, YEMEN

## Abstract

**Objectives:**

Research shows that in countries around the world, unregulated drug use and disorders are more prevalent amongst youth and young adults compared to general adult populations. Further, youth and young adults, including those in post-secondary settings, are increasingly experiencing harms from the global unregulated drug supply. Yet, population-specific harm reduction strategies are limited. This implementation science study explored the reach, effectiveness, adoption, implementation, and maintenance of a post-secondary campus-based drug checking service that was facilitated through intersectoral partnerships and run by students.

**Methods:**

This study used critical social theory and integrated knowledge translation. Data were collected from June to December 2023 through interviews and surveys that utilized the RE-AIM implementation science framework. One-time, individual semi-structured interviews were conducted with students (n = 4) who used drugs and/or the campus drug checking service and with program decision makers (n = 7) while student drug checking technicians (n = 6) completed online surveys. Data were analyzed using constant comparison.

**Results:**

Service reach was enhanced by a motivation to avoid consuming harmful substances but inhibited by stigma and fears of criminalization, particularly amidst uncertain academic repercussions. Participants reported that the presence and use of student-run drug checking services effectively reduced stigma on campus and the risk of harm for service users and their social networks. Adoption and implementation were facilitated by intersectoral partnerships and associated network expansion and resource sharing opportunities but challenged by inadequate infrastructure. The service was maintained through partnership agreements and individual commitments to engaging with drug checking services.

**Conclusion:**

This study contributes valuable insights regarding drug checking in post-secondary settings. These contributions are discussed in the context of young adult health and post-secondary environments, demonstrating how post-secondary institutions might help to overcome challenges in the implementation and delivery of campus drug checking services. The sustainability of such services requires supportive policies, enhanced accessibility, and related evaluations.

## Introduction

In countries around the world, studies show that unregulated drug use and disorders are more prevalent amongst youth and young adults compared to the general adult population [1–4]. This pattern is present in Canada, where a 2019 study showed that past-year unregulated drug use was reported amongst 14% of people aged 20 to 24 years, compared to 3% of people aged 25 years and older [2]. Similar statistics were seen in the United Kingdom (UK) in 2024, where 16.5% of people 16 to 24 years of age reported having used an unregulated drug in the previous year, compared to 9% of people 16 to 59 years of age [1]. As such, young people may be at risk of experiencing harm from unexpected contaminants or adulterants that are increasingly present in the unregulated drug supply (e.g., illicit opioids, benzodiazepines) [5]. When someone does not know what they are consuming (e.g., unexpected contaminants or adulterants), they cannot adequately prepare for the effects and protect themselves from potential harm. Such harm is evident in the United States (US), where, from 1998 to 2018, there was a 760% increase in polysubstance-involved deaths amongst people 13–25 years of age [6]. Similarly, in British Columbia (BC), Canada, there was a 406% increase in the annual unregulated drug death rate amongst people aged 19–29 years between 2014 and 2024 [7].

Young adults represented by these statistics include post-secondary students, who have been found to engage in different substance use behaviours than young adults who are not enrolled in post-secondary studies [8,9]. For example, in Jordan and the UK, researchers have found that post-secondary students have a higher prevalence of unregulated drug use than their non-student peers [10,11]. Further, research shows that after entering post-secondary environments in the UK, students are more likely to increase their use of, and their role in supplying, unregulated drugs [12,13].

It follows then that harm reduction interventions are increasingly being called for and implemented in post-secondary settings, but there remains a need for effective interventions that support the health of students who use unregulated drugs [14–20]. In the context of unregulated drug use, harm reduction strategies like drug checking can reduce the risk of consuming unexpected contaminants and adulterants. Drug checking is used in many settings worldwide to provide personalized harm reduction information about the contents of service users’ drugs, monitor drug supply trends [21], and facilitate engagement with health and social services [22,23]. Studies have found that drug checking effectively reduces the potential for harm for people who use drugs (PWUD) and their communities, including by preventing drug poisonings (e.g., overdoses), in festival and community settings [24,25]. Moreover, drug checking utilizes technologies that are found in many post-secondary chemistry and computer science labs, and this intervention integrates the skills taught in health and social services programs [21]. While this context provides an opportunity for students to engage in peer-based drug checking services, to our knowledge, drug checking in post-secondary settings has not been explored.

Amidst the rise in unregulated drug death rates for young adults, it is critical to explore feasible strategies that will reduce their risk of harm. Using the RE-AIM implementation science framework [26] (Table 1), we sought to evaluate the **r**each, **e**ffectiveness, **a**doption, **i**mplementation, and **m**aintenance of an intersectoral (university, health authority, community) post-secondary campus-based drug checking service in Kelowna, Canada.

**Table 1:** Definitions for domains of the RE-AIM implementation science framework as applied in a study of campus-based drug checking services.

RE-AIM domain	Definition
Reach	Who was using or not using the campus drug checking service, and why.
Effectiveness	The impact of the campus drug checking service on individuals and their quality of life.
Adoption	The settings, decision makers, and drug checking technicians who took up the service and their reasons for doing so.
Implementation	Facilitators and barriers for maintaining fidelity to drug checking guidelines and the program model.
Maintenance	How the drug checking service became part of university infrastructure and how individuals sustained their engagement with the service.

Definitions are adapted from Glasgow et al. (2019) [27].

## Methods

### Setting

In response to increasing deaths from BC’s unregulated drug supply [7], over 35 drug checking sites have been established in the province since 2016 [27] under a class exemption from section 56(1) of Canada’s Controlled Drugs and Substances Act [28]. Interest in drug checking spread to one BC university in 2019, when activists hired a non-profit organization from a nearby city to provide drug checking services at a 700-person campus party, without seeking permission from university administrators. Activists strategically mass-advertised the service 48 hours in advance, pushing administrators to quickly approve the service. Despite some initial reluctance, university administrators were pleased with the outcomes of the drug checking service.

Subsequently, university administrators supported the implementation of a student-run **ha**rm **r**eduction **t**eam (HaRT) to develop and disseminate resources (e.g., educational materials, naloxone, fentanyl test strips) [29] for the ~ 12,000 person student population [30]. According to a 2022 undergraduate survey at this university, half the respondents lived in housing on or near campus [31] and 16% of respondents reported using unspecified substances other than alcohol or cigarettes to relieve stress [32]. To coordinate HaRT (e.g., training, supervision, service operations, research, communications), the university hired a registered nurse, who was a doctoral student at the time (LA), for ~10 hours a week. Interdisciplinary undergraduate and graduate HaRT student staff (e.g., healthcare, social work, sciences, arts) shared ~33 hours a week and received training in trauma informed practice [33] and harm reduction. Additionally, HaRT was informed by experts, including PWUD, from the university, community, and the regional health authority.

Valuing HaRT students’ science and healthcare skills, the health authority initiated a contract with HaRT in 2020 to provide drug checking services through community organizations (e.g., overdose prevention sites, non-profit organizations) [29]. This contract included the provision of an Attenuated Total Reflectance Fourier Transform Infrared spectroscopy instrument (FTIR) that could be used to detect components present in a drug sample at a concentration of ~5% or greater [34]. FTIRs are widely used for their portability, relative affordability (~$60,000 CAD), speed, and the ability to return a sample to a service user as the instrument does not use up the sample [22,34,35]. These instruments are often used alongside benzodiazepine and fentanyl immunoassay strips for their ability to detect related metabolites [36]. Drug checking with FTIRs has been described at length in the literature [34,37–42]. While formal chemistry education lends itself to FTIR operations, harm reduction service providers have used FTIRs to provide meaningful drug checking results when they receive training and operations support from chemists or adjacent experts [22,34,35]. HaRT students who wanted to become FTIR drug checking technicians completed online modules and 30 hands-on training hours followed by an exam through the BC Centre on Substance Use’s [36] (BCCSU) drug checking program, which facilitates drug checking technician training, data stewardship, and operations.

HaRT was permitted to use the FTIR outside of community drug checking hours to provide campus-based drug checking services for the university community [43,44] and the university subsidized staffing costs for the campus-based service. The university campus-based drug checking service is the focus of this paper.

From 2021 to 2024, HaRT drug checking technicians, who were all students, provided campus-based drug checking services from September to April each year, closing for study breaks (e.g., reading week, holidays). The service was available two evenings a week in the first academic year and one evening a week in subsequent years to accommodate HaRT’s capacity and community FTIR access. HaRT offered drug checking in private and semi-open spaces in central student service areas (e.g., student health clinic, an integrated health space adjacent to the pub, a wellness office in a residence building) as well as at student union parties. HaRT students, including drug checking technicians, aimed to facilitate a welcoming and de-stigmatizing atmosphere by playing music, hanging lights, and displaying colourful harm reduction resources and education materials. Student service users were welcomed by HaRT students whether the service users had a sample for drug checking, were seeking resources, or wanted to engage in dialogue about harm reduction. Service users were not required to provide any identifying information.

Following BCCSU drug checking guidelines [36], drug checking technicians began their analysis by informing service users how drug checking worked, including technology limitations, and asking: (1) what they expected the sample to be; (2) if they had tried it yet, and; (3) if they had tried it, if there was anything unexpected about their response to the drug. Each service user then provided a ~ 2 mg sample that student drug checking technicians analyzed using FTIR spectroscopy alongside benzodiazepine and fentanyl immunoassay strips. During the ~ 10 minute analysis process, service users could observe the analysis, receive naloxone training, or review harm reduction resources with other HaRT students. If service users did not want to wait, they could choose to receive their results via text or e-mail. Service users who remained present could choose to have their sample returned to them or disposed of. On campus, most service users expected their samples to be psychedelics (Table 2).

**Table 2:** Number and proportion of expected sample types submitted to a university campus-based drug checking service.

Expected substance type	Academic year			Total
	September 2021 to April 2022	September 2022 to April 2023	September 2023 to April 2024	
Depressants^A^	28 (57.1%)	1 (3.2%)	4 (9.8%)	33 (27.3%)
Psychedelics^B^	14 (28.6%)	17 (54.8%)	26 (63.4%)	57 (47.1%)
Stimulants^C^	2 (4.1%)	8 (25.8%)	6 (14.6%)	16 (13.2%)
Unknown/other^D^	5 (10.2%)	5 (16.1%)	5 (12.2%)	15 (12.4%)
Total	49 (100%)	31 (100%)	41 (100%)	121 (100%)
Results				
Did not match expectation	17 (34.7%)	6 (19.4%)	15 (36.6%)	38 (31.4%)

A. Depressants: alprazolam, diazepam, fentanyl, oxycodone, phenibut, down. ^B.^Psychedelics: LSD, DMT, MDMA, MDA, Ketamine, 4-AcO-MiPT; Ketamine; 2C-B; 4-AcO-DMT; 5-MeO-DiPT. ^C.^Stimulants: methamphetamine, cocaine, speed, lisdexamfetamine. ^D.^Other: ivermectin, tadalafil.

### Positionality and philosophy

The lead author for this research is a white cisgender woman in her 30s with lived experience as a post-secondary student who used drugs. She is also a registered nurse and co-founder and former coordinator of HaRT. The research team sought to understand drug checking experiences according to participants’ own health and career goals, employing critical social theory and harm reduction as they embraced concepts of bodily autonomy and emancipation from societal structures [45–48]. This team of diverse decision makers, drug checking technicians, and PWUD facilitated integrated knowledge translation by informing all aspects of the study [49,50].

### Sampling and recruitment

Ethics approval was obtained from the Interior Health and University of British Columbia’s Okanagan Behavioural Research Ethics Boards [H23-00085] [H22-00720]. Recruitment for the study occurred from May 1^st^, 2023, to December 5^th^, 2023. Eligible participants had to be 18 years of age or older, speak and understand English, and be: (1) a student who used drugs and/or HaRT’s campus-based drug checking service; (2) a HaRT decision maker, or; (3) a HaRT drug checking technician. These populations were included for their different, critical perspectives on the reach, effectiveness, and feasibility of the intervention [51]. Recruitment occurred using purposive and snowball sampling [52,53] with the goal of recruiting four to six students who used drugs and/or HaRT’s campus-based drug checking service, six to nine decision makers, and six to nine technicians. The study was advertised through posters, social media, and e-mails.

### Data collection and analysis

For this study, quantitative data was embedded within a qualitative design [54]. Using guidelines from the RE-AIM website [55], researchers developed interview guides and surveys (Table 3) that inquired about each RE-AIM domain (Table 1) as they related to the drug checking service. Prior to initiating interviews and surveys, participants reviewed plain language consent documents and provided written or survey-response indication of consent as well as verbal consent at the start of each interview recording. From June to September 2023, decision maker and student participants completed interviews in person or over a secure video platform, lasting an average of 39–46 minutes respectively. From November to December 2023, drug checking technician participants completed online surveys instead of interviews to address confidentiality preferences. Data were stored according to university security requirements.

**Table 3:** Types of data collected for a post-secondary drug checking study stratified by participant group.

Data type	Data sources	Participant group		
		Decision makers	Drug checking technicians	Students
Qualitative	Semi-structured one-on-one interviews^A^	x	–	x
	Open text responses in online surveys:			
	implementation science surveys^A^	–	x	–
	member checking surveys^B^	x	x	x
Quantitative	Demographics questionnaires^A^	x	x	x
	Likert scale questions in online surveys:			
	implementation science surveys^A^	–	x	–
	member checking surveys^B^	x	x	x

^A^Current employees could complete data collection during work hours and students could choose to receive a $30 (CAD) cash honorarium; ^B^Drug checking technician participants could choose to receive a 25$ (CAD) e-gift card honorarium and student participants could choose to receive a $20 (CAD) cash honorarium.

Quantitative data were analyzed using basic calculations in Microsoft Excel for descriptive statistics (e.g., totals, percentages for demographics, one Likert scale question in the drug checking technician survey) [56]. Qualitative data were uploaded into NVivo and organized into RE-AIM categories [26] before undergoing thematic analysis using constant comparison [54,57]. Preliminary results were then shared for feedback through member checking surveys (Table 3).

## Results

Results are shared according to the RE-AIM categories (Table 1) [26]. Throughout this section, HaRT refers to the drug checking service, which is inclusive of student technicians, harm reduction student staff, and resources (e.g., naloxone, sterile supplies). To contextualize quotes, the participant’s group (e.g., decision maker [DM], drug checking technician [DCT]) is identified. ‘Students’ refers to all student participants who used drugs and/or the campus-based drug checking service, and within this group, students who used the campus-based drug checking service are referred to as ‘service user(s)’ while students who used drugs but did not utilize the campus-based drug checking service are referred to as ‘student(s)’ with an asterisk. Participants determined if they could be quoted, on what topics, and how to be identified. Student voices have been privileged in this paper.

### Study sample

The study sample (n = 17) included seven decision makers, six drug checking technicians, and four students who used drugs and/or the campus-based drug checking service (Table 4). Of these four students, two were service users, one had used a different drug checking service, and one did not report using drug checking services. All four students reported using psychedelics (DMT, LSD, MDMA, MDA, psilocybin, 2C-B), two reported using depressants (benzodiazepines, dilaudid, down, GHB, heroin, ketamine, methadone, percocet, sleeping pills, suboxone, Tylenol 3s), and one reported using stimulants (amphetamines, cocaine hydrochloride, cocaine base, methamphetamine, unspecified stimulants).

**Table 4:** Participant demographics^A^ from a study of a Canadian university campus drug checking service.

Demographic Variable	Drug checking technicians, decision makers ^B^ (n = 13)	Students (n = 4)
Age		
19-24	4 (33.3%)	2 (50%)
25-35	4 (33.3%)	2 (50%)
>36^B^	4 (33.3%)	
Gender^C^		
Woman	6 (50.0%)	1 (25%)
Man	4 (33.3%)	3 (75%)
Other^B^	4 (33.3%)	
Sexuality^C^		
Straight	6 (50.0%)	4 (100%)
2SLGBTQIA+^B^	6 (50.0%)	
Employment^C^		
Regular job full time	8 (66.7%)	2 (50%)
Regular part time job, temporary job, or self-employed^B^	6 (50.0%)	1 (25%)
Partner(s), family, friends, or student loans^B^	5 (41.7%)	2 (50%)
Income ($CAD)		
<$39,999^B^	5 (41.7%)	4 (100%)
>$40,000^B^	5 (41.7%)	
Ethnicity^C^		
Caucasian/white	10 (83.3%)	4 (100%)
Other^B^ (First Nations, Metis, Inuit, Inuk, South Asian)	5 (41.7%)	

^A^Eligible participants had to be 18 years of age or older, speak and understand English, and be: (1) a student who used drugs and/or the campus-based drug checking service; (2) a service decision maker, or; (3) a student drug checking technician. ^B^Categories were collapsed to better protect participant identities. ^C^Participants could select more than one response; the total responses may be greater than the number of participants.

### Reach

#### Advertising.

Three student participants reported hearing about the drug checking service through word-of-mouth, and Blair (student*) recalled seeing ‘cool’ posters featuring drug checking technicians they ‘recognized from school.’ Student participants thought advertising could be improved by showing drug checking as part of a normal daily routine, having professors endorse the service, and sharing about the drug checking service online in the comments section for events. They also believed humor would motivate people to share about drug checking service advertisements; Drew (student*) shared a potential ad tagline, ‘Want to get high? Might as well do it right.’

#### Criminalization and stigma.

Students said that fears of criminalization sometimes deterred themselves and other prospective service users, particularly at parties where there was a police presence. These fears were worsened by a limited understanding of how university substance use policies and repercussions differed from provincial legislation. Accordingly, students desired clear communications about service users’ rights.

Participants also shared that campus community members thought service users might be stigmatized. Drew (student*) commented on public stigma as an access barrier, stating, ‘someone’s addicted to drugs instead of food, and suddenly…they’re heavily stigmatized, ostracized.’ Additionally, drug abstinence campaigns in secondary schools had provoked ‘self-stigma’ that prevented Blair (student*) from accessing drug checking services and led students to feel like ‘bad people’ (Peyton, service user) for practicing harm reduction. However, participants believed that the campus location carried less potential for stigmatization than community settings ‘because there are people in the city, there’s no chance they would ever enter the [overdose prevention site]’ (Charlie, DM) for drug checking services.

Consequently, participants emphasized the importance of clean and comfortable drug checking service spaces where ‘people weren’t gawking’ (Avery, service user). Student and drug checking technician participants believed confidentiality could be enhanced if students could have their samples picked up or left in a ‘drop box’ (Quinn, DCT). Drew (student*) explained, ‘there’s this [guy who] does drugs to cope with the stresses…of being a first-year student…trying to work up the courage to go to [drug checking] but it’s like, “who am I going to see on the way?”’

### Accessibility

Service reach also depended on the timing of students’ unregulated drug use. Since drug checking only occurred ‘one day a week… [students] kind of have to plan’ (Avery, service user). ‘You have to [contact] a dealer and make sure [you see them] before the [drug checking] dates which is before the event’ (Peyton, service user). Such planning was difficult if students had relocated for school and had trouble ‘finding [their] footing’ (Blair, student*), or if they engaged in frequent or impulsive unregulated drug use. Blair (student*) said, ‘I want to do [a drug] when I buy it…I don’t really have time to wait’ and noted ‘when you’re dope sick you don’t really want to travel to test your drugs.’ Similarly, decision maker participants thought that the drug checking service reached the most students at parties because services were convenient to access at the time and place students planned to use drugs.

Therefore, all participants thought campus-based drug checking services should be available more often, including earlier in the day. Drew (student*) thought service hours should increase at strategic times, saying, ‘there’s some weekends that are coming up [when] everyone wants to get plastered… there’s the high season and the low season for drugs.’

Having a drug checking service on campus was considered convenient and critical since the university was in an isolated area of the city, most students did not have personal transit, and public transit was unreliable and time-consuming. Avery (service user) believed service locations should stay consistent, ‘not making it re-intimidating for people who might have already been uncomfortable and [had since] become comfortable.’ Still, participants wanted drug checking services to expand (e.g., mobile services, weekend nights downtown, fentanyl test strips freely available throughout campus) and for accessibility innovations to meet the needs of diverse student populations.

### Caring for the wellbeing of themselves and others

Service users accessed drug checking to mitigate the risks of unregulated drug use and to facilitate informed decision making for themselves and their friends. Peyton (service user) said, ‘I don’t want to have to choose between having the time of my life and losing my life’ as she was ‘very familiar with the fentanyl crisis.’ Likewise, Avery (service user) said it was ‘hard to get [drugs] that are what you want’ and ‘if I’m going to be doing stuff that’s potentially harmful, it’s good to mitigate the risks.’

### Effectiveness

Notably, decision maker participants thought more time was needed to understand the campus-based service’s impacts, due to COVID-19. They explained that it takes time for a drug checking service to establish trust within a community, and while HaRT’s drug checking service began in 2020, the first subsequent, full, in-person academic year began in September 2022.

### Destigmatization

Participants believed the drug checking service, including friendly student drug checking technicians, had helped to destigmatize harm reduction and conversations about unregulated drug use. Peyton (service user) shared, ‘knowing that I can go to my school and that there’s other students my age [checking] drugs to make sure that I’m safe and my peers are safe, it alleviates some of that shame that I have.’ Decision maker participants said that HaRT challenged assumptions about drug use, inspiring kindness and empathy, as participants described conversations with students who did not use drugs but who valued having drug checking available for their community.

### Health and wellbeing

HaRT reportedly had a positive impact on student participants’ wellbeing by providing personalized harm reduction information. Student participants described how, prior to HaRT’s drug checking service, their unregulated drug use was often accompanied by anxiety, fearing unanticipated effects. Since the campus drug checking service was implemented though, they felt less anxious and better able to care for their health. Avery (service user) reflected that HaRT was ‘where a lot of my knowledge around [drugs] that I’ve tried comes from, or I should say the informed knowledge…that I can back up.’ Even when student participants did not consume drugs, knowing their friends checked their own drugs led to more positive social experiences where participants felt ‘a lot more comfortable,’ could ‘have a better time’ (Drew, student*), and ‘worry a bit less’ (Avery, service user).

Avery (service user) thought ‘it’s good to have [campus drug checking services]…because people are going to use drugs whether or not there’s a [safe] supply.’ Certainly, student participants recalled disposing of their drugs after receiving unexpected drug checking results, preventing potential harm. Peyton (service user) said the drug checking service ‘alleviated the risk of losing [her] life.’

### Impacts beyond campus

The impacts of the drug checking service extended beyond campus as student participants reported sharing their drug checking results and newfound harm reduction knowledge (e.g., administering naloxone, lower risk drug use) with others. Peyton (service user) explained her motivations for disseminating information, saying, ‘we all did [drugs] in high school…with the future generation…we want [harm reduction resources] to be known.’ Inspired by HaRT, student participants had told their friends without access to drug checking services about at-home drug checking modalities and motivated their community workplaces to promote naloxone training.

Engaging with the drug checking service also led student and drug checking technician participants to seek out harm reduction career opportunities. This interest was reciprocated; Jan (DM) recalled a local non-profit that sought to hire HaRT students because HaRT ‘philosophically created good harm reduction people.’

### Adoption

#### Policies.

While the university publicly espoused HaRT, decision maker participants reported a perceived lack of buy-in from university administrators and an absence of drug checking policies. These participants believed administrators were concerned about liability risks (e.g., handling unregulated drugs, impacts of drug checking results) amidst an absence of provincial drug checking service standards. Given the seemingly insecure nature of the university’s commitment to the drug checking service, participants, particularly community and health authority decision makers, were hesitant to engage with the university’s drug checking service.

Drug checking technician participants expanded on how the lack of provincial drug checking service standards (e.g., staff scope, wages, workplace policies) was an adoption barrier. They expressed that it was especially challenging to know and work within the scope of their role. Consequently, they sometimes took initiative in ways where they unknowingly lacked the skills to execute their ideas, resulting in team conflict about responsibilities and dynamics.

#### Intersectoral partnerships.

The intersectoral partnerships reportedly facilitated applied public health research and resource sharing (e.g., technology, grants, education). Further, these relationships extended professional and service user networks, including by increasing the capacity of the harm reduction workforce. Drug checking technician participants especially valued learning from health authority and community experts, including PWUD, and the opportunity to apply classroom learning to a health intervention. These intersectoral relationships motivated decision maker and drug checking technician participants to adopt and advocate for the service.

#### Team infrastructure.

Drug checking technician participants described feeling valued and supported by HaRT, citing the team-based nature of the ‘great program’ (Jai, DCT) and ‘positive environment’ as ‘a big motivator’ (Quinn, DCT). However, HaRT’s limited funding translated to ‘inadequate compensation and hours’ (Quinn, DCT). Likewise, participants expressed guilt for taking time off, believing it would impact the availability of the drug checking service that week. To foster staff retainment, drug checking technician participants recommended higher wages, more hours to ‘feel more equipped’ (Koa, DCT) with their skills, and more ‘opportunities for career growth’ (David, DCT) through leadership roles. Additionally, participants identified that the whiteness of HaRT students might be a barrier to service adoption and uptake, and drug checking technician participants wanted to increase strategies to be more inclusive of Indigenous, Black, and other people of colour.

### Implementation

#### University environment.

Decision maker participants commented that academia was skilled at envisioning and evaluating interventions, but less experienced with program implementation and operations. Relatedly, they believed HaRT was limited as a university group that was not anchored within a harm reduction organization, where they otherwise might have a larger network of staff to assist with drug checking operations. Additionally, university funding stipulations meant that drug checking technicians *had* to be students, leading to staff turnover (e.g., graduation, quarterly class schedule changes). As such, decision maker participants valued having a program coordinator to support staff. However, they believed the frequent training was a drain on resources and that the coordinator’s subsequent pursuit of funding opportunities limited their availability to support service delivery.

#### Drug checking technician training.

Most drug checking technician survey respondents (n = 5) indicated feeling equipped to provide drug checking services and described taking initiative to pursue advanced training. Drug checking technician participants identified HaRT’s model of hiring students into harm reductionist roles first to solidify their ‘understanding of the language and ethical considerations’ (Jai, DCT) and slowly ‘training [drug checking technicians] over time [as] a good strategy’ (David, DCT).

#### Drug checking process.

Service operations were reportedly supported by a health authority drug checking leader, the BCCSU, and university student health managers. Drug checking technician participants solicited the expertise of these and other partners (e.g., community advocates, online drug checking network) to inform service operations. For sample analyses, in addition to instruments, drug checking technicians used ‘context or info from the person getting their drugs checked’ (Koa, DCT).

Decision maker and student participants found drug checking technicians and the drug checking service easy to engage with. Avery (service user) thought staff were ‘professional, and they had the medical services energy, it wasn’t judgemental in there at all’ and ‘they were super informed.’ Alongside drug checking, student participants described accessing harm reduction supplies and education (e.g., naloxone training), valuing that drug checking technicians often identified as PWUD. Still, student and decision maker participants suggested that HaRT integrate more healthcare professionals for drug-related health queries. Decision maker participants thought that staff should remain consistent and be complimented by students in experiential education roles (e.g., practicums, internships).

### Maintenance

#### Integration into the university.

The drug checking service was integrated into the university through funding, space provision, and public and institutional communications. However, decision makers worried that without policies to fortify service provision and access, HaRT would continue to have insufficient resources (e.g., funding, staffing) leading to inadequate capacity to: 1) produce data demonstrating the program’s value; 2) advocate for the drug checking service, and; 3) enhance drug checking service accessibility and subsequent engagement. Therefore, they believed the lack of policies made the drug checking service vulnerable to termination.

Additionally, memorandums of understanding had been established between intersectoral partners. However, decision maker participants desired clearer delineation regarding the boundaries of roles (e.g., service delivery, oversight, liability), research activities, and resource sharing. They also wanted more transparency on the value of the program through regular reports including engagement and expenditures data.

#### Individual maintenance.

When asked how likely it was that participants would continue to engage with the drug checking service, all participants responded positively. Koa (DCT) shared that they ‘love [their] job very much’ and Jan (DM) said ‘a hundred percent I will support this program.’ Student participants ‘deemed [drug checking] worth it…even if [they had] to travel, take a little bit of time, do a little bit of extra planning’ (Peyton, service user). Drew (student*) planned to ‘get [his] buddies to…collect their [drugs]’ and ‘test it for them.’ Further, Avery (service user) shared that drug checking had become a ‘common thing’ saying, ‘friends don’t take [drugs] that they don’t test now.’

#### Status of the Drug Checking Service.

In June 2024, university administrators prematurely terminated the health authority drug checking service contract. Consequently, the university forfeited access to the FTIR and facilitation of community drug checking services was transitioned to community-based groups. However, HaRT purchased an FTIR in February 2025 and advocacy efforts renewed university administrator support for campus-based drug checking services.

## Discussion

The present study drew on the perspectives of decision makers (n = 7), student drug checking technicians (n = 6), and post-secondary students who used drugs and/or the campus-based drug checking service (n = 4; Table 4) to evaluate the implementation of this service. The service’s reach was impeded by limited hours as well as fears of criminalization and stigma, but students were motivated to access the drug checking service to prevent harm from unregulated drugs. Certainly, the drug checking service helped to destigmatize and enhance harm reduction practices and knowledge for service users and their social circles. While the feasibility of the drug checking service was challenged by insufficient infrastructure and policies, drug checking technicians and decision makers valued the dynamic intersectoral team and associated opportunities. This study appears to be the first to evaluate drug checking in a post-secondary environment, offering valuable insights to inform future research as well as the development and sustainability of similar interventions.

In BC, over 2,700 people 19–29 years of age have died from unregulated drug use since 2015 [7]. This population includes students in post-secondary settings, where substance use stigma is prevalent [15,58,59]. Certainly, stigma was identified in the present and previous studies as a service access barrier for PWUD [60–62]. However, in the present study, students’ experiences of stigma, anxiety, and their risk of unregulated drug use harms were all offset by the presence and use of the student-run campus-based drug checking service. The importance of these findings might be further understood using the theory of set and setting, wherein a positive mindset and environment are ‘one of the first actions which can be undertaken to reduce drug harms’ (Hartogsohn, 2017, p.14) [63]. As such, we believe this is the first study to demonstrate that campus-based drug checking services may be a feasible strategy to respond to unregulated drug harms *as well as* stigma for some young adult populations. Further research is needed on drug checking service implementation and infrastructure in these settings, including exploration of the wellbeing implications for larger sample sizes and diverse student populations (e.g., racialized, 2SLGBTQIA+ students).

Past studies demonstrate that the impact of drug checking services extends beyond service users, as results are often shared with social circles [64,65]. As such, in Larnder et al.’s (2021) [66] study of third-party service users, they contend that only measuring the number of service users and samples does not capture the reach and impact of drug checking services. This finding is particularly relevant in youth and young adult populations, who have been found to exert influence on one another’s substance use behaviours [67,68] in a manner that may promote healthy subcultures [69]. Likewise, service users in the present study reported sharing about drug checking results and resources. Accordingly, drug checking services for these populations are likely to extend the influence of this intervention, including in decreasing drug-related harms.

Further, HaRT may have helped increase the capacity of the overburdened harm reduction field [70–73] by training students to become harm reductionists – individuals working to reduce the negative health, social, and legal impacts of drug use, laws, and policies [47]. However, the present findings show that training efforts were impeded by student transitions (courseloads, graduation) and insufficient funding. These challenges are not addressed by current drug checking service guidelines, meant for community [28] and event [74] contexts. Certainly, drug checking guidelines must diversify without enforcing strict standards that might become implementation barriers for the diversity of drug checking service contexts and the associated intersectoral partnerships [21,23,75–77]. Additionally, post-secondary experiential education models (e.g., practicums, internships) that include drug checking technician training should be implemented and evaluated, including exploration of strategies to offset staff turnover and longitudinal evaluations of career trajectories and impacts on the harm reduction field.

Finally, the present study corroborates literature showing how inadequate drug checking policy makes the intervention vulnerable to fluctuating political interests, funding, and adoption hesitancy [25,64,78]. Participants in the present study believed the absence of university drug checking policies stemmed from fears of liability, despite legal drug checking service exemptions in BC [28] and guidance from expert bodies [79,80]. Notably, HaRT research partner Dr. Lukas Bichler commented in a presentation about the present drug checking service that he had worked with nuclear and radioactive materials, yet ‘working with [unregulated] drugs can sometimes be more complicated in terms of access paperwork, security, and safety’ [81]. Certainly, literature demonstrates how stigmatizing perceptions of liability risk may be more based on historical societal norms than actual risk [58,82,83]. This study makes important contributions to the lack of literature on post-secondary drug checking services and may inform enhanced policy support, funding, and infrastructure for post-secondary drug checking services. More research is needed with larger sample sizes on drug checking policies and liability conceptions in post-secondary settings.

### Strengths and Limitations

This study sought to address a gap in the literature regarding post-secondary drug checking services. The RE-AIM framework [26] facilitated the management of multiple objectives and data sources for a complex intervention, resulting in clearly articulated pragmatic considerations. Study methods facilitated rich descriptions of participant experiences [56] that integrated knowledge translation helped to represent and address [49].

However, this study has some limitations. Data that did not fit the RE-AIM domains [26] were excluded from analysis, although participants confirmed that the findings reflected their experiences. Further, the small sample size lacked diversity amongst student participants and may not reflect experiences of the larger student body. Additionally, this study reflects a unique context, and the generalizability of findings may be limited. It is possible that social desirability bias [84] influenced the data, although researchers aimed to mitigate this risk by having peer interviewers. Since analysis was conducted by the HaRT coordinator at the time, there was the risk of bias in the interpretation and communication of findings. Rigorous ethics procedures were followed to address these concerns (e.g., de-identifying data, limiting access to interview locations) and their perspective facilitated valuable insights into the data.

## Conclusion

This study provided novel insights regarding post-secondary drug checking services, and the potential for far-reaching impacts. Participants characterized this intervention as having positively affected the health of student service users and their social circles, including by preventing unregulated drug use harms. While decision makers and drug checking technicians were challenged by the absence of drug checking policies, they were bolstered by supportive and resourceful intersectoral relationships. The sustainability of post-secondary drug checking services requires supportive program policies as well as enhanced service accessibility and related evaluations. Such strategies are critical to explore amidst the worsening harms for young adults who use unregulated drugs.
